# *Ex Vivo* and *In Vivo* Mice Models to Study *Blastocystis* spp. Adhesion, Colonization and Pathology: Closer to Proving Koch's Postulates

**DOI:** 10.1371/journal.pone.0160458

**Published:** 2016-08-10

**Authors:** Sitara S. R. Ajjampur, Chin Wen Png, Wan Ni Chia, Yongliang Zhang, Kevin S. W. Tan

**Affiliations:** Laboratory of Molecular and Cellular Parasitology, Department of Microbiology and Immunology, National University of Singapore, 5 Science Drive 2, Singapore, 117545; Agency for Science, Technology and Research—Singapore Immunology Network, SINGAPORE

## Abstract

*Blastocystis* spp. are widely prevalent extra cellular, non-motile anerobic protists that inhabit the gastrointestinal tract. Although *Blastocystis* spp. have been associated with gastrointestinal symptoms, irritable bowel syndrome and urticaria, their clinical significance has remained controversial. We established an *ex vivo* mouse explant model to characterize adhesion in the context of tissue architecture and presence of the mucin layer. Using confocal microscopy with tissue whole mounts and two axenic isolates of *Blastocystis* spp., subtype 7 with notable differences in adhesion to intestinal epithelial cells (IEC), isolate B (ST7-B) and isolate H (more adhesive, ST7-H), we showed that adhesion is both isolate dependent and tissue trophic. The more adhesive isolate, ST7-H was found to bind preferentially to the colon tissue than caecum and terminal ileum. Both isolates were also found to have mucinolytic effects. We then adapted a DSS colitis mouse model as a susceptible model to study colonization and acute infection by intra-caecal inoculation of trophic *Blastocystis* spp.cells. We found that the more adhesive isolate ST7-H was also a better colonizer with more mice shedding parasites and for a longer duration than ST7-B. Adhesion and colonization was also associated with increased virulence as ST7-H infected mice showed greater tissue damage than ST7-B. Both the *ex vivo* and *in vivo* models used in this study showed that *Blastocystis* spp. remain luminal and predominantly associated with mucin. This was further confirmed using colonic loop experiments. We were also successfully able to re-infect a second batch of mice with ST7-H isolates obtained from fecal cultures and demonstrated similar histopathological findings and tissue damage thereby coming closer to proving Koch’s postulates for this parasite.

## Introduction

*Blastocystis* spp. have been attributed to be the most common protists detected in human faecal samples in numerous studies globally [1,2]. Reported prevalence rates of this gastrointestinal pathogen are higher in developing countries (up to 63–100%) [3,4] than developed countries (0.5–24%) [2]. In developing countries, risk of acquiring *Blastocystis* spp. has been associated with intra familial transmission, lack of piped water supply, poor maternal education and zoonotic transmission [3,5,6]. *Blastocystis* spp. have also been implicated as a cause of irritable bowel syndrome [7,8] and urticaria [9,10]. In addition to human infections, *Blastocystis* spp. have been demonstrated in a wide range of hosts including insects, reptiles, birds and small mammals and livestock including cattle and pigs [11,12]. Based on SSU rRNA sequencing, *Blastocystis* spp. are now considered to be a species complex of 17 different subtypes (ST1-17) that exhibit some degree of host specificity [12–14]. Among these, ST1 to ST9 have been reported in humans with most infections associated with ST3 and ST1 followed by ST2 (mostly seen in South America) and ST4 (high prevalence in Europe and Australia) [2]. ST7 although not exhibiting a high prevalence worldwide, has been reported from several countries in Asia and Africa including Egypt, Nepal, Pakistan, Malaysia and Singapore [2].

However, despite high prevalence rates, the link between *Blastocystis* spp. and gastroenteritis has been disputed due to multiple reasons. One reason is that blastocystosis manifests as non-specific gastrointestinal symptoms of flatulence, vomiting, abdominal pain, and diarrhea and so remains indistinguishable from other causes of diarrhea [15]. Other reasons include a large proportion of reportedly asymptomatic infections [16] and a significant number diagnosed as ‘co-infections’ [17,18] or simply not being reported. The role of subtype has also been examined and although there have been some co-relations drawn between subtypes and symptoms, they are not conclusive due to the absence of appropriate epidemiological controls and differences in methodology [9,19,20]. Although *in vitro* studies with axenic cultures of ST1, ST4 and ST7 and animal studies with axenic isolates or purified cysts have clearly demonstrated pathogenic potential, the molecular and cellular basis of pathogenicity of *Blastocystis* spp. has not been fully elucidated [21,22]. Additionally, due to a lack of suitable animal models, Kochs’ postulates for this parasite have not been fulfilled till date [21].

The pathogenic potential demonstrated by this parasite includes the ability to damage the intestinal epithelium resulting in increased permeability both by inducing apoptosis [23] as well as by degrading tight junction proteins [23,24]. Both *in vitro* and *in vivo* studies have demonstrated the ability to induce a proinflammatory response with production of IL-8 and GM-CSF by human colonic epithelial cells [25], up regulation of IFNγ, IL-12 and TNFα mRNA in the colon of 3 week old Wistar rats infected with ST4 cysts orally [26] and presence of inflammatory infiltrates in the sub-mucosa of 2–6 week old BALB/c mice intra-caecally infected with axenic cultures of ST7 [27]. More recently, lysates of ST7 resulted in up regulation of IL1β, TNFα and IL6 in mouse intestinal explants and macrophages [28]. This pathogenic potential extends to excretory secretory products of the parasite. Cysteine proteases of *Blastocystis* spp. ST4 and ST7 were shown to cleave human secretory immunoglobulin A (IgA) [29]. A cell surface cysteine protease, legumain has been identified to be involved in a pro-survival role in *Blastocystis* spp. and may also be a potential virulence factor due to its ability to activate other proteases such as cathepsins [30,31].

In the gastrointestinal tract, adhesion of pathogens to the intestinal epithelial cells has been found to be a crucial early step in the pathogenic process [32]. This would help avoid removal due to peristalsis and enable colonization. Recent *in vitro* studies have shown that adhesiveness to Caco-2 cells was subtype dependent with ST7 being more adhesive than ST4 [33,34]. Significant intra-subtype differences were also observed, with isolate ST7-H being the most adhesive compared to isolates ST7-B, ST7-C, ST7-E and ST7-G [34]. This variation in adhesiveness also had a hierarchical correlation to virulence with more adhesive strains associated with greater degradation of tight junction proteins ZO-1 and occludin and resultant increase in intestinal permeability [34]. The intestinal epithelium *in vivo*, is however, not readily accessible due to the presence of the thick mucus layer with an outer, loosely packed layer and an inner, sterile layer that is continuously replenished [35,36]. In the large intestine, the most prominent component of the mucin layer is the heavily glycosylated muc2 [36]. *Blastocystis* spp. being non-motile, in order to contact and adhere to the intestinal epithelium, as an initial event, have to traverse this mucus barrier [34]. Histopathological studies in a mouse model have shown that the protist localizes to the lumen or on the mucosal edge of the caecum and colon along with deposits of mucin [27]. In rats, *Blastocystis* spp. result in chronic infections over several weeks [37,38]. Histopathology showed that the parasites remained in the lumen and lead to an increase in neutral mucin containing goblet cells in the colon [26]. In naturally infected pigs, parasites were seen in the lumen and on the mucosal surface in association with fecal matter and mucus [39,40]. While there have been a few descriptions in mice and naturally infected animals mentioned above, there have been no previous studies that examined the interaction of *Blastocystis* spp. with the intestinal mucin layer.

Several *ex vivo* models have been developed to create a more complex and physiologically relevant intestinal environment [41–43]. These models allow examination of host pathogen interactions and pathophysiological changes in the context of tissue architecture [43]. To extend previous studies on adhesion from *in vitro* models of IEC to more closely resemble the intestinal environment, we developed an *ex vivo* model using explant tissue from C57BL/6 mice that would allow the study of *Blastocystis* spp. interaction with the mucin layer. To examine whether adhesiveness then influenced colonization rates, we adapted a mouse model of acute infection. For the purposes of this study, two axenic isolates of *Blastocystis* spp., ST7 were used with notable differences in adhesion to IEC, isolate ST7-B and isolate ST7-H. We showed that adhesion to mucin and intestinal tissue explants and the ability to colonize mice has an intra-subtype variation similar to adhesion to IEC and could potentially explain the variability in reported association of *Blastocystis* spp. with gastrointestinal symptoms.

## Methods

### Ethics statement

*Blastocystis* isolates ST7-H and ST7-B were obtained from the Department of Microbiology collection at National University of Singapore (NUS). These human isolates were obtained from patients at the Singapore General Hospital in the early 1990s, before the Institutional Review Board was established in NUS. All samples were anonymized. The animal experiments were performed in accordance with the Singapore National Advisory Committee for Laboratory Animal Research Guidelines. The protocol (R13-5890) was reviewed and approved by the NUS Institutional Animal Care and Use Committee.

### *Blastocystis* spp. isolates

Axenic cultures of previously characterized *Blastocystis* spp. ST7-H and ST7-B originally recovered from symptomatic patients were used in this study. The two isolates were maintained in culture in pre-reduced Iscove's modified Dulbecco's medium (IMDM, Gibco) containing 10% heat-inactivated horse serum in an anaerobic jar (Oxoid) with an AnaeroGen gas pack (Oxoid) at 37°C [29,44].

### Mucin adhesion

*Blastocystis* spp. cells in log-phase, collected after 24 hours of culture, were washed twice with phosphate buffered saline (PBS) and stained with carboxy-fluorescein diacetatesuccinimidyl ester (CFSE; Invitrogen) for 15 minutes at 37°C at a final concentration of 20 M. The cells were then washed with PBS to remove the excessive stain. Porcine gastric mucin (PGM, Sigma) in a microtiter plate based assay described previously with some modifications was used [45]. Briefly, PGM diluted in PBS was coated on 96 well ELISA plates (Nunc Immunosorp) at a concentration of 100 μg/well overnight at 4°C. Control wells coated with PBS alone were also included. The plates were washed and blocked with 2% skim milk followed by washing. *Blastocystis* spp. CFSE stained cells were added at a concentration of 10^6^ per well and incubated anerobically at 37°C for 2 hours. The unbound cells were then washed and 1% SDS in 0.1M NaOH was added to lyse bound cells. The cell lysates were then mixed well and transferred to a black 96 well plate and fluorescence read at 485 nm. Adhesion was expressed as the percentage of fluorescence recovered after binding to mucin coated or PBS wells relative to the fluorescence of the cell suspension added to the wells. The fold change in adhesion to mucin coated relative to PBS coated wells for the two isolates was then compared.

### Explant harvest

Mice were purchased from the National University of Singapore (NUS) and housed in the ABSL2 clean animal facility. Explants for this study were obtained from 7–9 week old C57BL/6 mice. Animals were euthanized by CO_2_ inhalation and the intestinal tract dissected out and placed in 50 ml tubes with complete media comprising Dulbecco's modified Eagle's medium (DMEM) (HyClone) supplemented with 10% heat-inactivated fetal bovine serum (FBS) and 1% each of sodium pyruvate and MEM and Penicillin-Streptomycin (Gibco) at a final concentrations of 2,000 units/mL of penicillin and 2,000 mg/mL of streptomycin and immediately transported to the laboratory on ice. The tissue was then dissected into segments of distal colon, proximal colon, caecum and terminal ileum, opened along the mesenteric edge and intestinal contents removed. The segments were washed gently in cold Penicillin-Streptomycin and cut into bits measuring 1.5 cm x 1 cm. The explants were affixed onto 2% agarose layers in 6 well plates with the serosal surface facing down in prewarmed complete media with Penicillin-Streptomycin (Gibco) (S1A and S1B Fig). For all assays, more than one litter was used in order to avoid any litter-specific effects and at least 5 mice were tested for each condition.

### Explant adhesion assay

For each well, 5×10^7^ CFSE-stained live parasites diluted in complete media were added onto the mucosal surface and incubated for 2 hours in a humidified incubator with 5% CO_2_ at 37°C. After incubation, tissue bits were washed gently with sterile PBS and fixed with freshly prepared methacarn for 2 hours at 4°C. Fixed tissue was then permeabilized and blocked with buffer containing 3% BSA, 5% Normal Goat Serum and 1% Triton X in PBS. The explant tissue was then stained with H300 rabbit anti-MUC2 antibody (Santa Cruz) overnight at 4°C followed by secondary goat anti rabbit Cy5 (Life technologies) for 2 hours at 4°C. Tissues were then incubated with Hoechst 33258 (1:1000) to stain nuclei for 30 minutes. Whole mount tissues were placed on coverslips with flouromount (Sigma) and imaged with confocal microscopic examination (Olympus Fluoview FV1000, Olympus, Japan). Parameters for imaging were 20x magnification with 2x zoom, 10–12 z stack with 2μm slice thickness and at least 3 images per sample. Uninfected tissue controls were included for each mouse and tissue type (S1C Fig). Quantitative analysis of *Blastocystis* spp. adhesion to intestinal explant tissue was carried out using the spot counting tool in IMARIS defined as diameter of ≥4μm.

### Mucin secretion by Enzyme Linked Lectin Assay (ELLA)

Explants prepared as mentioned above were treated with 5×10^7^ live parasites and incubated for 1 hour and6 hours at 37°C. After incubation, the supernatants were collected, centrifuged and frozen at -20°C. Mucin levels in supernatants from treated and untreated explants were compared using an ELLA assay. Wheat germ agglutinin (WGA) binds to N-acetyl-b-glucosamine and has been used previously to detect intestinal mucins.Frozen supernatants were thawed and used to coat 96 well plates along with porcine gastric mucin standards as controls (Nunc immunosorp) overnight at 4°C. After washing with PBS-T, 100μl of wheat germ lectin conjugated to horse radish peroxidase (0.1 μg/ml) (Sigma) was added and incubated for 1 hour at room temperature followed by washing and addition of substrate ABTS (Sigma). Absorbance was read at 405 nm.

### *In vivo* model of colonization

C57BL/6 male mice aged 5–6 weeks were given 2% Dextran Sulfate Sodium Salt (DSS) in drinking water for four days followed by a recovery period of 5 days. At 7–8 weeks they were intra-caecally inoculated with 5×10^7^ live parasites and followed up for 3 days. Briefly, the mice were anaesthetized (Ketamine 75mg/kg + Medetomidine 1mg/kgIP) followed by a mid-line incision on the abdomen. The caecum was exteriorized and parasites suspended in 100–150 μl of saline were injected into the caecum with a 27G needle. The abdomen was then closed in 2 layers with sutures, anaesthesia was reversed (Atipamezole 1mg/kg SC) and the mice were given antibiotics (Enrofloxacin10mg/kg SC) and analgesic (Carprofen 5mg/kg SC) and followed up with daily fecal sample collection. Sham surgical controls were included with each batch and were given saline intra-caecally. On the third day, the mice were euthanized and the intestinal contents and tissue were harvested. Intestinal tissue was used to prepare Swiss rolls and fixed in 10% formalin overnight followed by processing for paraffin embedding and staining with hematoxylin and eosin, PAS at an external facility (AMPL, Singapore). Histological tissue scoring to assess intestinal tissue damage was carried out using a previously validated protocol [46]. Fecal samples and intestinal contents collected were cultured in Jones media [47] and followed up for up to 10 days to detect colonization. Cultures were scored as positive based on the presence of vacuolar forms (S2 Fig).

### Statistics

Comparisons between two groups were performed using non-parametric Mann-Whitney and Wilcoxon matched-pairs signed rank test. Comparisons between multiple groups were made using ANOVA test. GraphPad Prism version 6 for Windows (GraphPad, San Diego, CA) was used for analysis; p<0.05 was considered statistically significant.

## Results and Discussion

### *Blastocystis* spp. adhesion to explants is isolate dependant and shows tissue trophic effects

**Fig 1:**
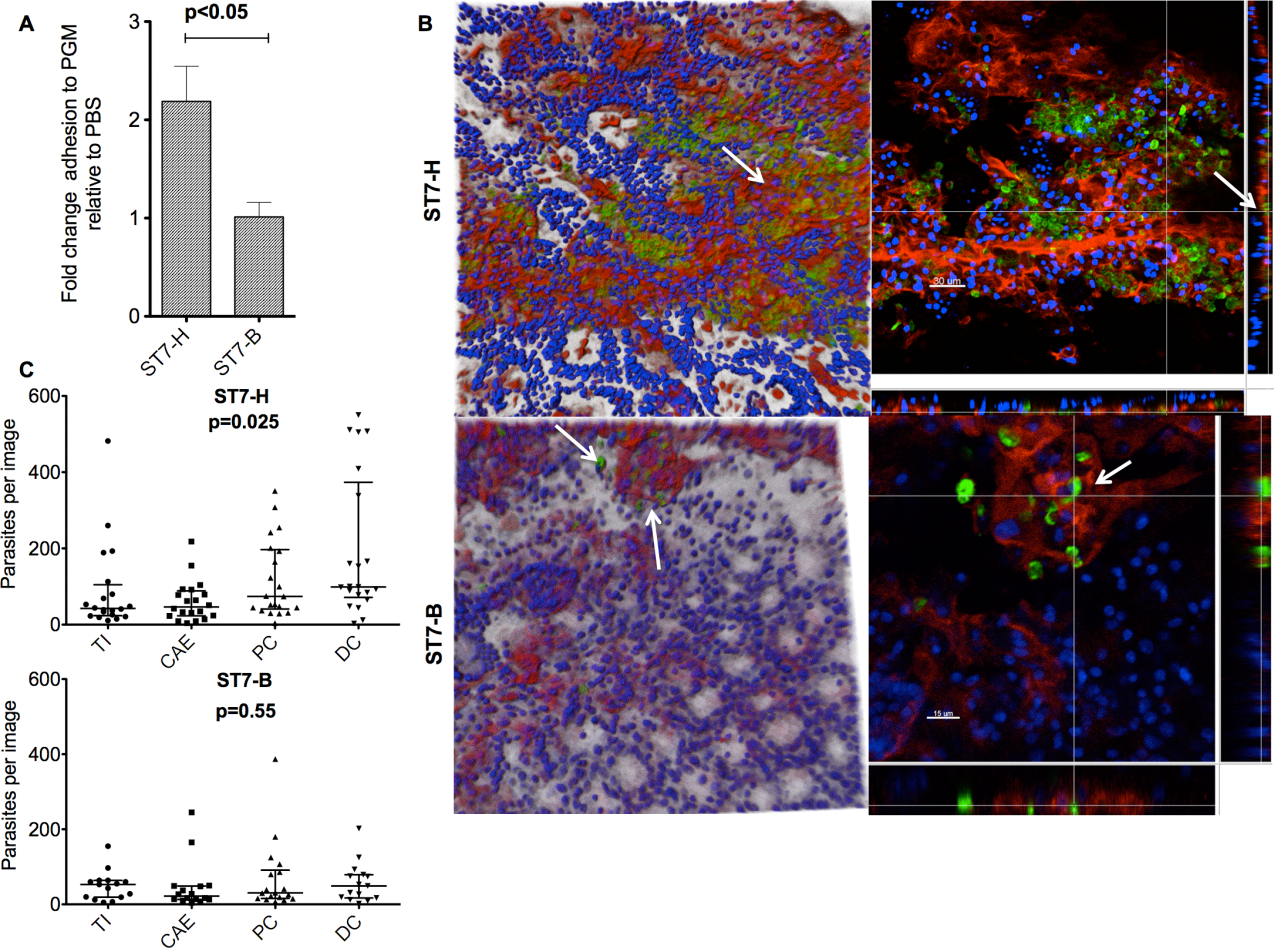
*Blastocystis* spp. adhesion to mucin on the surface of intestinal explants shows intra-subtype variation. (A) Bar graph represents fold change of *Blastocystis* spp. adhesion to porcine gastric mucin coated wells relative to PBS coated wells. ST7-H had significantly higher binding than ST7-B. Data representative of 3 independent experiments. (B) Confocal micrographs of mice distal colon incubated with *Blastocystis* spp. ST7-H and ST7-B showed binding of the parasites to the mucin layer of intestinal explants. Representative images from 5 mice explants are shown. Tissues were incubated with CFSE-stained *Blastocystis* spp. (green) and counterstained for muc2 (red) and Hoecht for nuclei (blue). (C) Quantitative assessment of *Blastocystis* spp. adhesion to intestinal explants showed intra-subtype variation and tissue trophic effects. Spot counting data for ST7-H and ST7-B incubated with terminal ileum, caecum, proximal colon and distal colon from at least 5 mice. Horizontal bars represent median and range.

Prior to experiments with an *ex vivo* model, a preliminary experiment on adhesion of the two isolates to commercially available porcine gastric mucin (PGM) was carried out. Using 96-well plates coated with PGM or PBS alone and incubated anaerobically with 10^6^ CFSE stained *Blastocystis* spp. cells, the bound cells were lysed and the proportion of fluorescence was determined. ST7-H showed a 2 fold increase in adhesion to PGM coated wells relative to PBS coated wells (Fig 1A) while ST7-B did not show any increase providing an indication of intra-subtype differences in adhesion to mucin.

When intestinal tissue explants from C57BL/6 mice were incubated with *Blastocystis* spp. isolates ST7-H and ST7-B on the mucosal surface, confocal microscopy of whole tissue mounts revealed that the parasites co-localized mostly to the mucin layer (Fig 1B). For both isolates, clusters of fluorescent cells were seen in the muc2 antibody-stained mucin-rich areas on the surface of the explant. Adhesion was seen in explants derived from all segments of the large intestine from terminal ileum to the distal colon. Quantitative assessment of binding to intestinal explants using spot counting software however, showed that this binding was not uniform across the four tissue types tested (Fig 1C). ST7-H showed a preferential binding of parasites to the proximal and distal colon compared to the caecum and terminal ileum (p<0.05) but ST7-B did not show similar tissue tropism and was found to bind more uniformly across all intestinal segments (p = 0.55). When the 2 subtypes were compared, ST7-H showed increased binding to the proximal (range, IQR, 74, 41–197) and distal colon (99, 71–373) compared to ST7-B (30, 15–91 and 49, 17–79 respectively) (p<0.05 and p<0.05 respectively).

This study has shown that adhesiveness to mucin and intestinal tissue has intra-subtype variation and this mirrors previous findings on adhesion to IEC [34]. ST7-H isolate binds to more than one type of mucin as the mucin in commercial PGM is predominantly muc5 while that on mouse large intestine is predominantly muc2. This is the first study to quantitatively document adhesion to mucin by this gastrointestinal protist. Adhesion to mucin has been considered to enhance infectivity of a pathogen by providing protection against other anti-microbial agents and leading to downstream changes in gene expression priming the pathogen for infection or invasion of the epithelial layer [48–50]. For *Blastocystis* spp. being non-motile and non-invasive, mucin may provide a crucial niche environment in the large intestine for early survival and colonization of the host. Interestingly, we also documented tissue tropism in the more adhesive isolate with greater adhesiveness to the colon. This increased adhesion to colon is a novel finding and may be a potential virulence factor allowing increased and preferential binding to the colon and colonization *in vivo*.

### *Blastocystis* spp. degrades mucin from all segments of the large intestine

**Fig 2:**
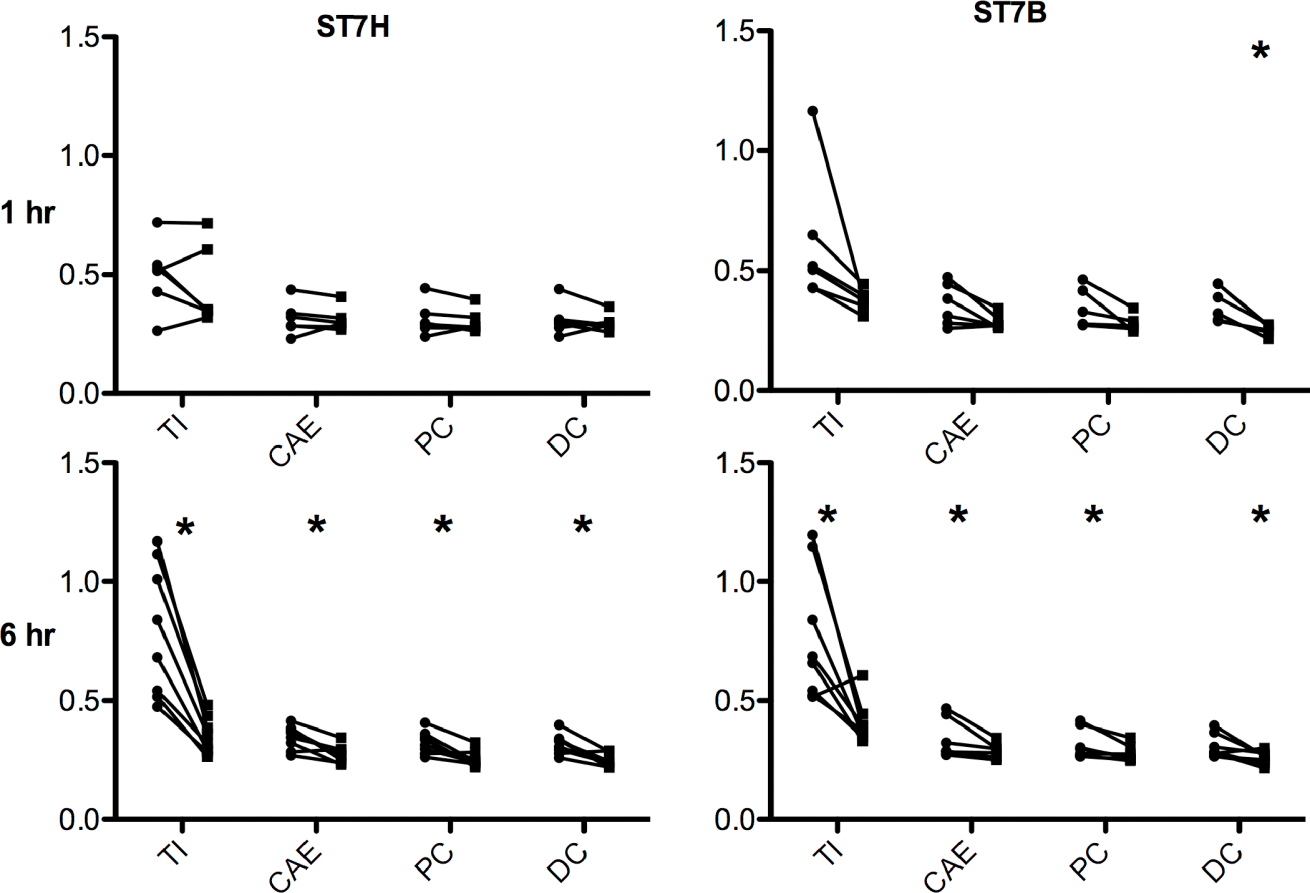
Mucinolytic effects of *Blastocystis* spp. Levels of mucin in the supernatants of paired infected and uninfected explant tissue. Significant mucin degradation in the infected explants at 6 hours was seen for both isolates but only ST7-B showed degradation of colonic mucin at 1 hour. Mucin levels were estimated by ELLA and the data is representative of explants from at least 5 mice each. *p<0.05.

Explants from the same segment of the intestine were divided longitudinally and incubated with either complete media (uninfected) or complete media containing *Blastocystis* spp. (infected) for 1 and 6 hours. When mucin levels in these paired samples were estimated by ELLA using a WGA- conjugate, we showed that by 6 hours both ST7-H and ST7-B had degraded a significant amount of mucin compared to the uninfected tissue controls (Fig 2). Interestingly, at the 1 hour time point only ST7-B showed mucinolytic activity and there was a significant reduction in the distal colon.

Intestinal parasites have been shown to affect the mucin barrier by multiple mechanisms including penetrating or degrading mucin [51,52], inducing hypersecretion or emptying of goblet cells, or secretagogue effects [53], or altering the quality or nature of mucin secreted resulting in a more penetrable barrier [50,53]. It is also not surprising for a pathogen to both adhere to and degrade mucin in the gastrointestinal tract. Other pathogens that have been described to have similar effects include *Candida albicans*, *Helicobacter pylori*, *Shigella* and *Vibrio cholera* [35,54]. This study shows that even at an early time point of 1 hour *Blastocystis* spp. tends to have a mucinolytic effect rather than a secretagogue effect. Both isolates were found to degrade mucin at 6 hours. The levels of mucin in the terminal ileum control tissue were found to be higher than in controls from other tissues. This could in part, be due to a lower level of bacterial microflora in the ileum compared to caecal and colonic tissues that may have contributed to mucin degradation to some extent [55]. Nevertheless, there was a significant difference between control and infected tissue showing that *Blastocystis* spp. co-incubation resulted in mucin degradation.

### *Blastocystis* spp ST7-H is a better colonizer of C57/BL6 mice and causes greater tissue damage

**Fig 3:**
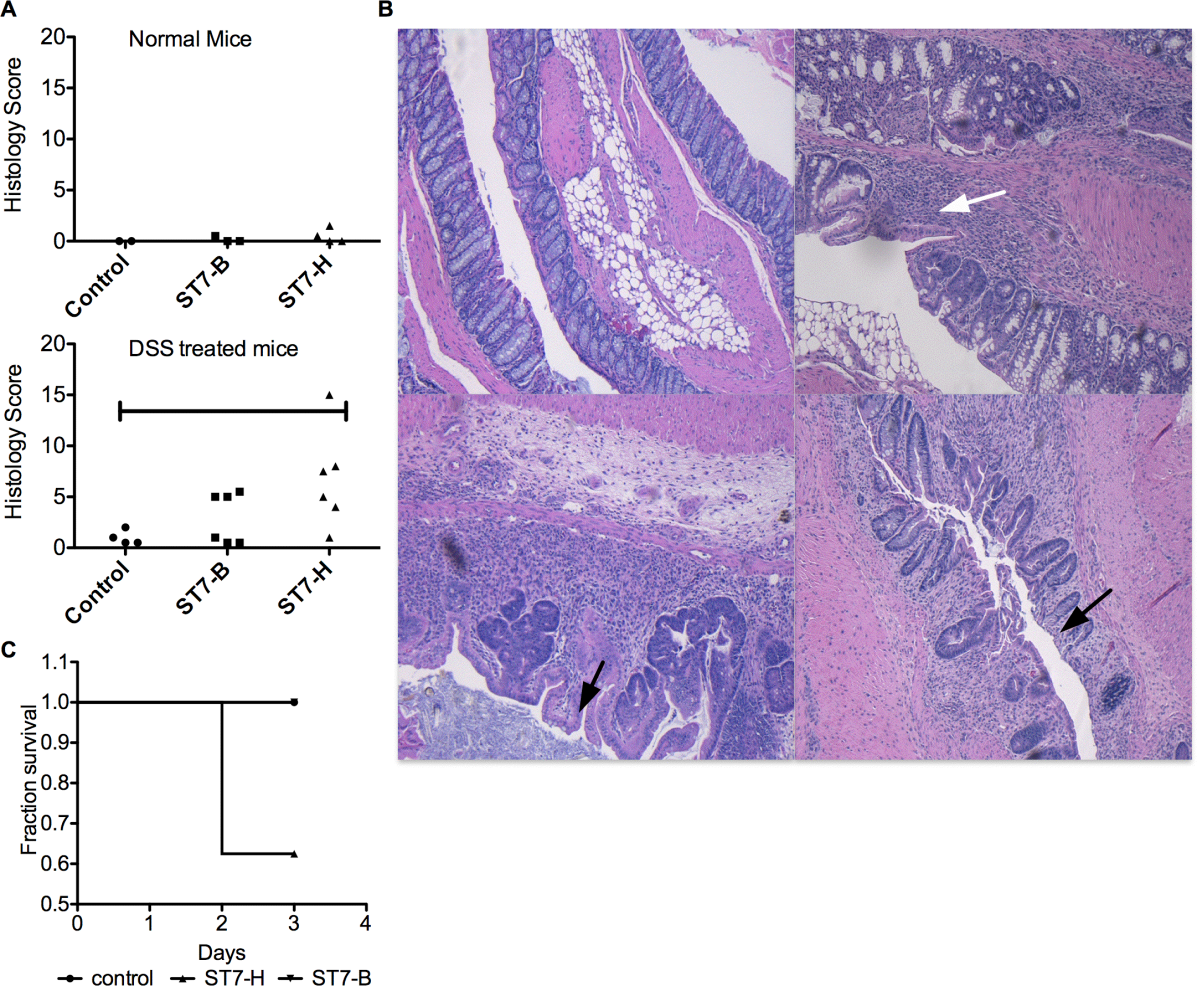
Histopathological examination of mice infected with *Blastocystis* spp. (A) Tissue damage assessed by histological scoring showed little evidence of damage in normal mice. Significant damage was seen in DSS-treated mice infected with ST7-H compared to sham surgical controls. * p<0.05. (B) Representative histological images of tissue damage in distal colon of infected DSS-treated mice. clockwise from top left (1) DSS treated mouse control (2) & (3) ST7-H infected mice with moderate crypt loss (black arrow) and inflammatory cell infiltration (white arrow) (4) ST7-B infected mouse with moderate crypt loss (black arrow) and occasional inflammatory infiltration (C) Survival analysis showed ST7H had higher mortality (p = 0.17) while ST7B (p = 1) was similar to sham surgical controls but this did not approach statistical significance.

In order to establish a model for colonization and acute diarrhea, 7–8 week old C57/BL6 mice were inoculated intra-cecally with *Blastocystis* spp and followed up for 3 days. This however did not lead to successful colonization. These mice shed *Blastocystis* spp. for only 24 hours (Table 1A) and showed little to no evidence of tissue damage compared to sham surgical controls (Fig 3A). Mice have however, been previously demonstrated to be refractory to *Blastocystis* spp infection [27]. A DSS colitis model was then adapted using a lower dosage (2% DSS) and shorter duration of treatment (4 days) to induce mild colitis and was used for infection after a 5 day recovery period. When these mice were intra-caecally innoculated with *Blastocystis* spp., they were found to develop acute diarrhea with loose to watery feces and shed parasites for 2–3 days (Table 1B). Colonization rates for ST7-H (6/7 mice) were higher than for ST7-B (3/6). Infection with ST7-H also resulted in increased duration of shedding for up to 3 days. Sham infected mice did not develop diarrhea and had formed fecal pellets on all days till sacrifice. Increased susceptibility of the DSS treated mice compared to normal mice also points to the important role of mucin in this model. DSS treatment results in early biophysical changes in the mucin barrier resulting in increased penetrability to commensal bacteria. The resulting colonization of the “inner” sterile layer by bacteria is thought to result in development of inflammation and colitis [56,57]. In this study, the resulting colitis and inflammation increased susceptibility to *Blastocystis* spp. in an otherwise refractory animal model.

**Table 1:** Fecal and intestinal cultures using Jones media from (A) normal mice, (B) DSS-treated mice and (C) Parasites recovered in culture were used to re-infect DSS-treated mice.

		Feces			Intestinal Content
**(A) Normal mice**		Day 1	Day 2	Day 3	Day 3
Control	C1	0	0	0	0
	C2	0	0	0	0
ST7-B	B1	+	0	0	0
	B2	-	0	0	0
	B3	0	0	0	0
ST7-H	H1	+	0	0	0
	H2	-	0	0	0
	H3	+	0	0	0
	H4	+	0	0	0
**(B) DSS treated mice**					
Control	C1	0	0	0	0
	C2	0	0	0	0
	C3	0	0	0	0
	C4	0	0	0	0
ST7-B	B2	+	+	0	0
	B3	0	+	0	0
	B4	0	0	0	0
	B5	++	0	0	0
	B6	-	0	0	0
	B7	-	0	0	0
ST7-H	H1	0	+	++	++
	H2	0	X	X	X
	H3	+	+	0	0
	H4	+	0	0	0
	H6	-	++	X	0
	H7	+	0	0	0
	H8	-	+	X	X
**(C) DSS treated mice infected with Jones culture**					
Flora^a^	CF1	0	0	0	0
	CF2	0	0	0	0
ST7-H^b^	H/J1	+	+	0	0
	H/J2	++	0	++	++

- indicates no sample collected, X indicates no sample collected as animal died, and 0 indicates Jones cultures remained negative after 10 day follow up.

^a^Control mice were infected with bacterial flora recovered in Jones cultures to ascertain no pathological effect.

^b^Mice infected with Jones cultures of 5x10^6^ *Blastocystis* spp.

When overall tissue histological scores were compared, ST7-H caused significantly more damage to the intestine when compared to the DSS colitis controls (Fig 3A, p = 0.032) than ST7-B (p = 0.37). This additive damage of the tissue included disruption of crypt architecture, goblet cell loss (mostly in the distal colon) and presence of leukocytes in the lamina propria (Fig 3B). *Blastocystis* spp. were observed within the lumen and at the surface of the epithelial cells. No invasive forms were found. When histological scores were compared for individual sections of the large intestine, both in the caecum and in the distal colon, ST7-H caused significant tissue damage compared to surgical controls (p = 0.014 and 0.047 respectively) than ST7-B (p = 0.14 and 0.32 respectively). When a survival analysis was carried out (Fig 3C) although ST7-H-infected mice had higher mortality rates (p = 0.17) than ST7-B-infected mice (p = 1) which were similar to the sham surgical controls, the difference was not statistically significant.

**Fig 4:**
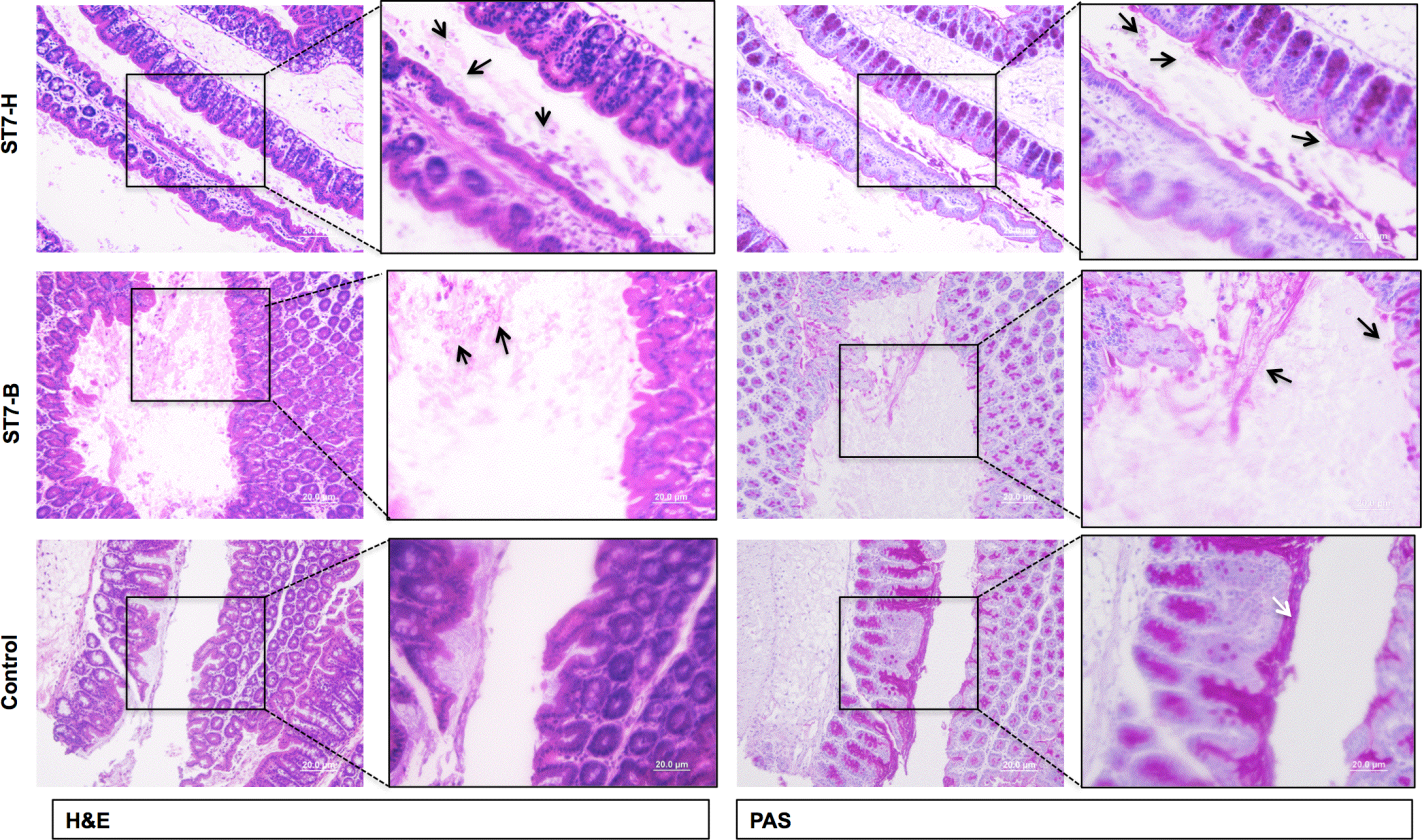
*Blastocystis* spp. remains luminal and binds to mucin *in vivo*. Representative histology of colon loops from DSS treated C57BL/6 mice (starved overnight to minimize intestinal contents) inoculated with 5x10^7^*Blastocystis* spp. ST7-H or ST7-B or saline (controls) for 1 hour *in vivo*, gently washed and preserved in methacarn. *Blastocystis spp*. were found only in the lumen and mucus layer for both isolates. Black arrows indicate luminal and mucin bound parasites and the white arrow in the control tissue shows well-preserved PAS stained inner mucin layer.

To further substantiate the luminal nature of the parasite, colon loop experiments were carried out in 3–4 mice in which ST7-H and ST7-B cultures were injected into ligated colon loops with another loop acting as a control. The mice were starved overnight to minimize intestinal contents. After a 1hour incubation during which the mice remained anesthetized, the colon loops were harvested, washed gently and fixed in methacarn. Further histological processing included both hematoxylin and eosin as well as periodic acid Schiff stain. These sections (Fig 4) showed that the parasites remained luminal and were closely associated with the mucin layer. The association with mucin was both on the surface of the epithelial cells as well as free clusters of mucin seen in the lumen. Interestingly, the mucin layer was also found to be better preserved in the colon loops injected with saline, indicating that even within a short incubation period, the parasite begins to disrupt the mucin layer.

### Koch’s postulates

**Fig 5:**
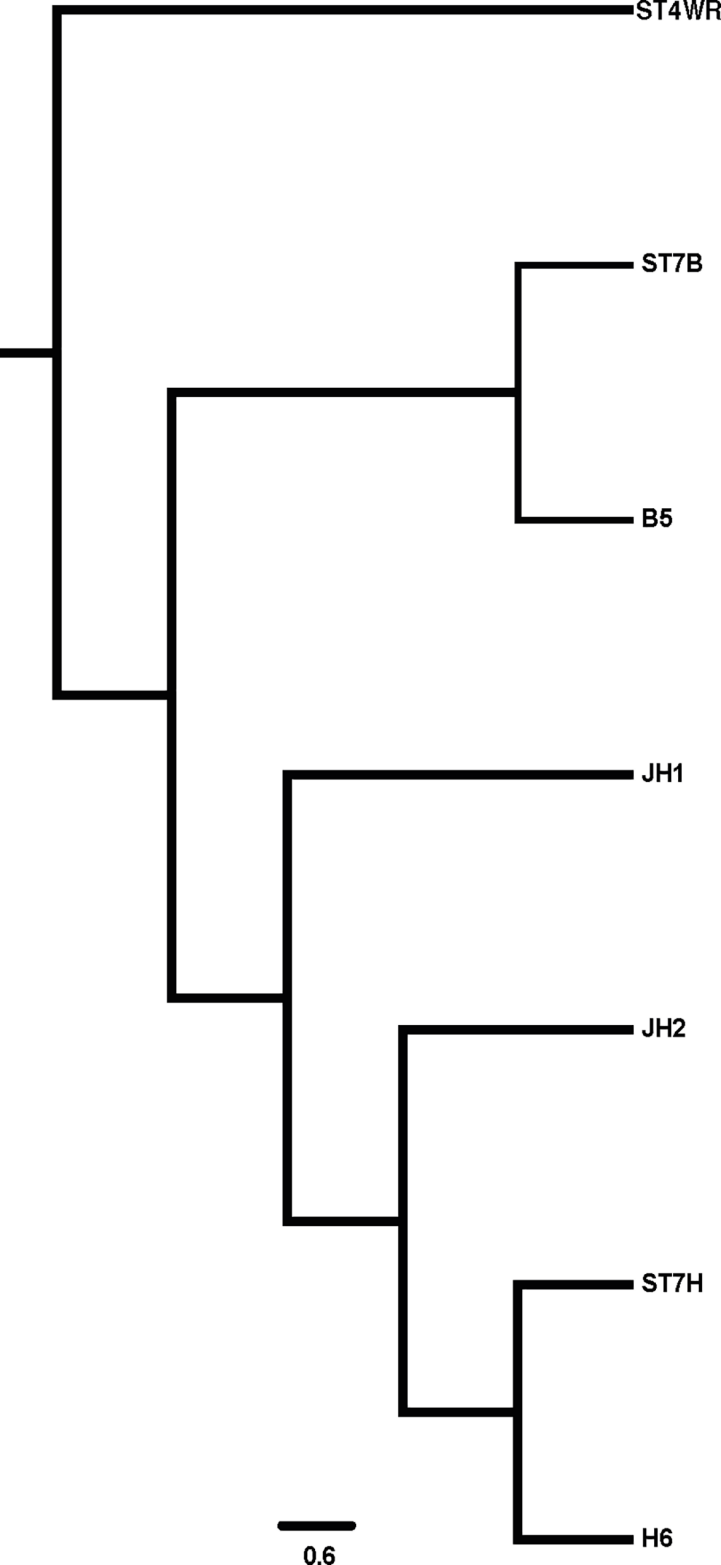
Koch's postulates applied to DSS treated mouse model. Phylogenetic analysis of isolates by PCR-sequencing of SSU rDNA. ST4-WR1, ST7-B, ST7-H represent axenic isolates maintained in the laboratory, B5 and H6 are isolates recovered by Jones culture of mice fecal samples and JH1 and JH2 are isolates from mice re-infected with H6.

Using ST7-H isolates recovered from Jones cultures of DSS treated mice feces, we were able to successfully re-infect 2 mice (Table 1C). The mice were colonized by ST7-H even though the intra-caecal inoculum was decreased by 1 log (5x10^6^) compared to the previous axenic cultures. Control mice were inoculated with the associated bacterial flora grown in the xenic cultures. Histopathologic results showed tissue damage in mice that were inoculated with ST7-H cultures (scores 5.5 and 2) while mice that were inoculated with the associated bacterial flora alone had little to none, similar to the sham surgical controls (both had scores of 1). The parasites were found in the lumen and along the mucosa with no invasive stages. The fecal culture from these mice also grew ST7-H in Jones media for 2–3 days. DNA was extracted from a subset of fecal samples and SSU rRNA PCR [58] and sequencing carried out. Phylogenetic analysis showed that the isolates obtained from Jones cultures were identical to the axenic ST7-H and ST7-B isolates used (Fig 5). This is the first study to show that isolates recovered from mice with acute infection and tissue damage resulted in symptoms in another batch of mice. These symptoms could be attributed exclusively to *Blastocystis* spp. as mice infected with fecal culture-associated bacterial flora alone remained asymptomatic. This is an important step towards proving Koch’s postulates and further studies with a longer time line and more isolates will help prove this more convincingly. However, in the light of a recent study showing the effect of *Blastocystis* spp. on diversity of the microbiome in colonized individuals [59], a more detailed characterization of the microflora in animal models will be required to determine the role in disease.

Some of the limitations of this study include the small number of isolates tested and that we only used isolates from single subtype. Further studies using isolates from multiple subtypes as well as a more detailed characterization of both mucin binding and degradation will need to be carried out as well a binding to other glycoconjugates. Effect of mucin binding on *Blastocystis* spp. gene expression also needs to be investigated. The *in vivo* model can also be extended to include a longer duration of infection to study resolution of infection and role of immune response. In a recent study, *Blastocystis* spp. infected individuals were found to have a greater diversity in their gut microflora

**Fig 6:**
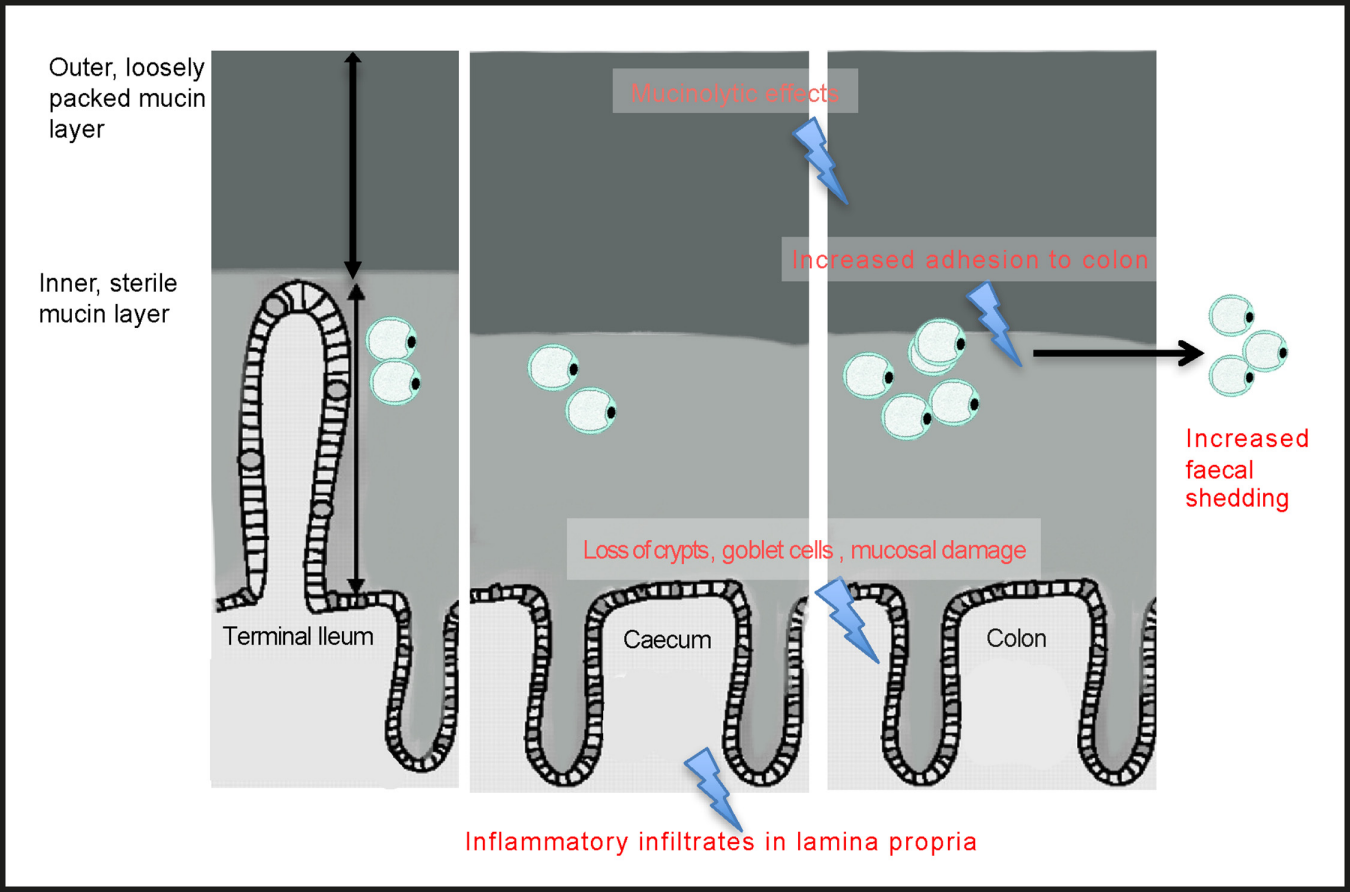
Model of adhesion and colonization: *Blastocystis* spp. are luminal, mucin binding and mucinolytic. ST7-H is more adhesive than ST7-B and shows tissue tropism to colon. Increased adhesiveness results in better colonization in DSS treated susceptible mice with a longer duration of shedding and increased tissue damage and inflammation.

In summary, in this study we established an *ex vivo* model to study interaction of *Blastocystis* spp. with the intestinal epithelium and mucin and adapted a DSS colitis *in vivo* model to study acute infection and colonization of mice. Beyond this study, this *ex vivo* model can also be used to characterize effect of *Blastocystis* spp. on immune response and other host factors. We showed that intra-subtype variation in binding to IEC was mirrored in binding to mucin and intestinal explants. This increased adhesiveness also translated to better colonization and increased tissue damage and could help potentially explain the variability associated with symptoms in clinical isolates (Fig 6). The novel finding of tropism for colonic tissue in the more adhesive and more virulent isolate could also explain this variability. Both the *ex vivo* and *in vivo* models as well colon loop experiments also demonstrate the luminal, non-invasive nature of the parasite. *Blastocystis* spp. was found to adhere to both surface and luminal mucin and increased binding to mucin facilitated colonization and infection. This also validated previous studies in mice, rats and naturally infected pigs that found the nature of the parasite to be luminal and not invasive. Additionally, we showed that susceptibility to *Blastocystis* spp. could be induced by inducing prior inflammation in the host intestine with DSS treatment. This suggests a role as an opportunistic pathogen for *Blastocystis* spp. and could explain the increasing link with IBS. Most importantly, we were also able to show that on re-infection with isolates from fecal cultures, we were able to replicate similar tissue damage paving the way to proving Kochs’ postulates for this controversial pathogen.

## Supporting Information

S1 Dataset Data from assays on porcine gastric mucin and tissue explant adhesion, ELLA and tissue damage and survival in mice. (XLSX)

S1 Fig Preparation of explants for whole mount confocal imaging. (A) Tissue explants on agarose beds (B) Terminal ileum villi visualized under light microscope prior to co-incubation with *Blastocystis* spp. (C) Confocal image of control caecal tissue stained withfor muc2 (red) and Hoecht for nuclei (blue) showing inner rim of goblet cells with with mucin.
(TIFF)

S2 Fig Representative image of vacuolar *Blastocystis* spp. cells. *Blastocystis spp*. seen in Jones culture of fecal samples and intestinal contents of infected mice.
(TIFF)
