# Supplementary figures and images for: Ex Vivo and In Vivo Mice Models to Study Blastocystis spp. Adhesion, Colonization and Pathology: Closer to Proving Koch's Postulates
Source: PLoS One. 2016 Aug 10;11(8):e0160458. doi: 10.1371/journal.pone.0160458 (PMC4979897; doi:10.1371/journal.pone.0160458)

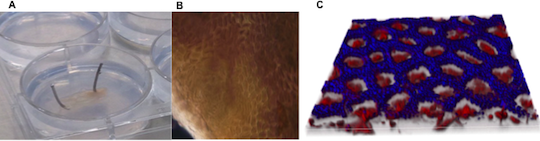

Supplement: S1 Fig — (A) Tissue explants on agarose beds (B) Terminal ileum villi visualized under light microscope prior to co-incubation with Blastocystis spp. (C) Confocal image of control caecal tissue stained withfor muc2 (red) and Hoecht for nuclei (blue) showing inner rim of goblet cells with with mucin. (TIFF) [file pone.0160458.s002.tiff]

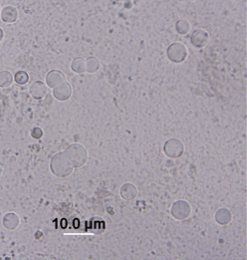

Supplement: S2 Fig — Blastocystis spp. seen in Jones culture of fecal samples and intestinal contents of infected mice. (TIFF) [file pone.0160458.s003.tiff]
